# Drug resistance mechanism and reversal strategy in lung cancer immunotherapy

**DOI:** 10.3389/fphar.2023.1230824

**Published:** 2023-09-19

**Authors:** Yishi Xu, Yaqing Liu, Yi Ge, Haozhe Li, Yi Zhang, Liping Wang

**Affiliations:** First Affiliated Hospital of Zhengzhou University, Zhengzhou, China

**Keywords:** lung cancer, immunotherapy, immune check inhibitor (ICI), drug resistance, reversal strategy

## Abstract

Among all malignant tumors, lung cancer has the highest mortality and morbidity rates. The non-small cell lung cancer (NSCLC) and small cell lung cancer (SCLC) are the most common histological subtypes. Although there are a number of internationally recognized lung cancer therapy regimens, their therapeutic effects remain inadequate. The outlook for individuals with lung carcinoma has ameliorated partly thanks to the intensive study of the tumor microenvironment and immune checkpoint inhibitors. Numerous cancers have been effectively treated with immunotherapy, which has had positive therapeutic results. Global clinical trials have validated that PD-1/PD-L1 inhibitors are effective and safe for treating lung cancer either independently or in combination, and they are gradually being recommended as systemic treatment medications by numerous guidelines. However, the immunotherapy resistance restricts the immunotherapy efficacy due to the formation of tumor immunosuppressive microenvironment and tumor mutations, and immunotherapy is only effective for a small percentage of lung cancer patients. To summarize, while tumor immunotherapy is benefiting an increasing number of lung cancer patients, most of them still develop natural or acquired resistance during immunotherapy. Consequently, a crucial and urgent topic is understanding and tackling drug resistance triggered by immunotherapy in lung cancer treatment. This review will outline the presently recognized mechanisms of immunotherapy resistance and reversal strategies in lung cancer.

## 1 Introduction

**FIGURE 1:**
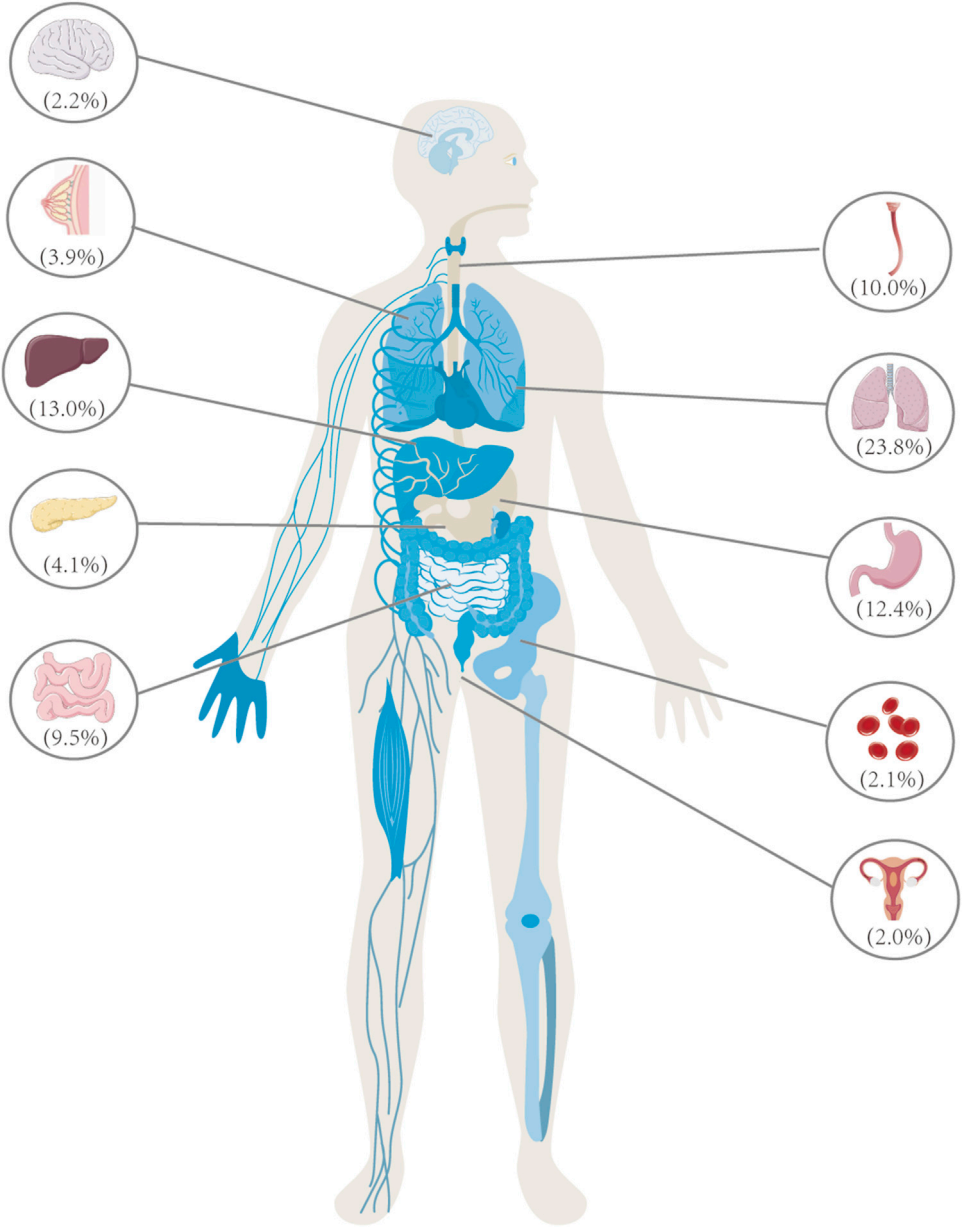
Lung cancer is the primary contributor to cancer-related mortality among individuals aged 50 and above, surpassing the total fatalities attributed to breast, prostate, and colorectal cancer. In the year 2020, China witnessed a total of 3 million deaths caused by cancer, with lung cancer being the leading cause, accounting for a significant proportion of 710,000 deaths, representing 23.8% of the overall cancer-related fatalities. In 2020, the leading cancer types in China were as follows: lung cancer (710,000 cases), liver cancer (390,000 cases), gastric cancer (370,000 cases), esophageal cancer (300,000 cases), colorectal cancer (290,000 cases), pancreatic cancer (120,000 cases), breast cancer (120,000 cases), nervous system cancer (70,000 cases), leukemia (60,000 cases), and cervical cancer (60,000 cases). These ten types of cancer are responsible for approximately 83% of the total number of deaths caused by cancer.

Most often, lung cancer is fatal and has a poor prognosis (Nooreldeen and Bach, 2021; Thai et al., 2021; Siegel et al., 2023). Patients with advanced lung cancer have a 5-year survival rate of less than 10% (Ning et al., 2021). Late-stage lung cancer can spread to other organs, most commonly the brain, bone, lymph nodes, and other areas, causing severe pain and possibly endangering patients’ lives. At the time of diagnosis, most NSCLC patients had metastases (Ko et al., 2021); among them, bone and brain are the most frequently metastatic locations. As the tremendous advances of various immune checkpoint inhibitors in hematologic tumors and melanoma develop, the gradual rise of immunotherapy brings new opportunities and challenges and is currently one of the primary focus areas for researchers (Horn et al., 2017; Wang et al., 2019; Reck et al., 2022; Xie et al., 2022). In contrast to conventional chemotherapy or targeted therapy, immunotherapy primarily regulates and enhances the function of the human immune system through advanced immunotherapy technology, relying on the recovery and improvement of autoimmunity to kill tumor cells (Szeto and Finley, 2019; Carlino et al., 2021). Immunotherapy approaches that are now in use include medication therapy (such as anti-PD1/PDL1 monoclonal antibodies, interleukin 2, interferon alpha, and so on), cell therapy, and tumor vaccines, among others. In theory, immunotherapy can treat all types of cancer patients. According to clinical experience, preoperative immunotherapy can fight for the chance of surgery, and reduce the recurrence rate after surgery (Forde et al., 2018; Lee et al., 2019; Reuss et al., 2020). In addition, postoperative immunotherapy can reduce the risk of recurrence after surgery by promoting physical recovery and killing residual tumor cells (Zhu et al., 2021; Chaft et al., 2022). The toxicity of radiation treatment can be mitigated and the effectiveness of radiotherapy medications can be improved by combining them with immunotherapy. Advanced cancer patients can benefit from immunotherapy, which can result in long-term survival from tumors (Ueta et al., 1998; Kato et al., 2019; Wang et al., 2022b) (Figure 1).

Immunotherapy, including cell Programmed Death 1 (PD- 1), Programmed Death- Ligand 1(PD-L1), and Cytotoxic T- Lymphocyte Antigen- 4 (CTLA-4) inhibitors, has been an effective treatment for lung cancer in the last few years. The integration of immunotherapy and chemotherapy has become the established initial treatment for advanced NSCLC lacking gene mutations. Several licensed medications, in accordance with clinical standards, are available for marketing and utilization in this context. However, research into immunotherapy for lung cancer has not stopped, and drug resistance remains a prevalent and serious clinical challenge that requires urgent attention. Moreover, multiple studies have indicated that individuals diagnosed with lung cancer exhibit a higher propensity for developing resistance to PD-L1 inhibitors in comparison to other patient cohorts (Schoenfeld and Hellmann, 2020; Schoenfeld et al., 2021; Passaro et al., 2022).

The definition of immune resistance is still not completely unified based on clinical manifestations and biological characteristics, and the cancer immunotherapy society’s classification of immune resistance modes is widely accepted: primary resistance, acquired resistance, and patients who relapse after stopping treatment for reasons other than toxicity. Primary resistance is characterized as the advancement of disease following a minimum of two cycles of immunotherapy within a time frame of no longer than 6 months. Secondary resistance refers to disease progression in patients who have seen clinical benefit or whose disease has been stable for more than 6 months (Rizvi et al., 2023). Progression or relapse in clinical practice might emerge as primary immunological resistance, mixed resistance, or acquired resistance, which can be difficult to recognize. This is due to the fact that we cannot fully explain the mechanism of immune resistance, and the diversity and complexity of biology, as well as the presence of hyper progression and spurious progression, make defining this mechanism more difficult (Cheson et al., 2016; Danylesko et al., 2021), and the different mechanisms of resistance will influence the formulation of follow-up treatments. According to current research, the mechanism of immunotherapy drug resistance may be correlated with changes in tumor internal factors (such as driver genes, tumor mutation burden) and the formation of tumor immunosuppressive microenvironment, which fails the immune system’s typical response, manifested as primary or secondary drug resistance. Based on this, further research into immunotherapy resistance prediction and reversal strategies following drug resistance is required (Schoenfeld and Hellmann, 2020). This article will also examine tumor internal elements and immunological microenvironment in immune resistance formation.

## 2 The current state of lung cancer immunotherapy

Lung cancer has the most somatic mutations and acquired treatment resistance (Schoenfeld and Hellmann, 2020). According to WHO pathological analysis (Marx et al., 2015), there are four distinct classifications of lung cancer, namely, small cell lung cancer (SCLC), lung adenocarcinoma, lung squamous cell carcinoma, and large cell lung cancer, the latter three of which can also be referred to collectively as non-small cell lung cancer (NSCLC). Furthermore, NSCLC and SCLC were detected through genetic testing; the latter is a collection of multi-gene abnormalities, with numerous driver genes cooperating (Oser et al., 2015; Hamilton and Rath, 2019). SCLC has early metastasis, predominantly multiple metastases, and a poor prognosis based on its pathological characteristics (Molina et al., 2008; Hamilton and Rath, 2019). The treatment of lung cancer has seen significant transformations over the past decade as a result of the introduction of immune checkpoint inhibitors (ICIs). Prior to more than a decade ago, terminal NSCLC and SCLC had a Median Survival Time (MST) of only 1 year, and platinum-based chemotherapy emerged as the primary therapeutic approach for these individuals. Due to their discovery and availability, immune checkpoint inhibitors have revolutionized lung cancer treatment, particularly NSCLC (Gadgeel et al., 2020). Immunotherapy has significantly improved NSCLC Patient Overall Survival (OS), whether they are adjuvant or neoadjuvant (Paz-Ares et al., 2018). When advanced disease is treated with immunotherapy, the overall survival rate for SCLC patients is minimally improved compared with NSCLC patients (Hamilton and Rath, 2019). Suppressing PD-1/PD-L1 or CTLA-4 in NSCLC and SCLC at different stages has been the subject of multiple clinical investigations.

### 2.1 Immunotherapy advancements in NSCLC

The Impower trial is a phase III clinical trial that encompasses a cohort of 1,280 patients diagnosed with stage IB-IIIA non-small cell lung cancer (NSCLC) and who have undergone complete surgical removal of their tumors. 1,269 of them were treated with cisplatin-based chemotherapy (cisplatin plus pemetrexed, docetaxel, gemcitabine, or vincristine), then 1,005 were given either 16 rounds of Atezolizumab or the best supportive care (BSC). The primary goal of the trial was disease-free survival (DFS), with Overall Survival (OS) serving as the secondary outcome of the study. There was a significant difference between how well Atezolizumab and BSC worked to cure people in stage II-III A who had PD-L1 positive (TPS 1%) cancer. According to the most recent OS data in 2022, the most significant improvement in OS (76.8% vs. 67.5%) was reported in stage II-III A patients with PD-L1 expression1%. Atezolizumab-treated patients with PD-L1 TPS 50% and no EGFR/ALK + saw a 17.3% increase in OS over BSC-treated patients (84.8% vs. 67.5%) (Felip et al., 2021).

Besides PD-L1 inhibitors, PD-1 inhibitor Nivolumab paired with chemotherapy has made significant advances in neoadjuvant lung cancer therapy (Forde et al., 2022). Nevertheless, CTLA-4 inhibitors are yet to be evaluated in the treatment of NSCLC. Researchers found that dual immunosuppressant therapy with or without chemotherapy is superior to monotherapy based on preliminary results from studies of Checkmate-9LA and Checkmate-227 (Hellmann et al., 2019; Paz-Ares et al., 2021; Brahmer et al., 2023). The creation of dual immune checkpoint inhibitors is an additional significant development in recent research. Among them, AK104 is the first bispecific antibody to target PD-1/CTLA-4 to enter clinical trials worldwide, is significantly more effective than monotherapy with PD-1/PD-L1 and CTLA-4, and effectively reduces toxic side effects associated with monotherapy (Pang et al., 2023) (Table 1).

**TABLE 1:** Trials using combination immunotherapies for NSCLC.

Trial identifier	Target	Treatment strategy	Phase	End point
NCT04638582	PD-1	Pembrolizumab + Platinum-pemetrexed	Phase 2	ctDNA、OS、DFS
NCT05565378	PD-1	Dostarlimab + GSK4428859A	Phase 2	ORR、OS、PFS
NCT04581824	PD-1	Dostarlimab + Chemotherapy	Phase 2	ORR、OS、PFS
NCT05085028	PD-1	Pembrolizumab	Phase 3	OS、PFS
NCT04475939	PD-1	Pembrolizumab + niraparib or placebo	Phase 3	OS、PFS
	PARP			
NCT03377023	PD-1	Nivolumab + Ipilimumab + Nintedanib	Phase 1	MTD、ORR
	VEGF		Phase 2	
NCT03800134	PD-L1	Durvalumab/placebo + platinum-based chemotherapy	Phase 3	pCR、EFS
NCT05221840	PD-L1	Durvalumab + Oleclumab or Monalizumab/placebo	Phase 3	PFS、OS
	CD73			
	NKG2A			
NCT04171284	PD-1	SCT-I10+ Docetaxel/Placebo	Phase 3	OS
NCT03866980	PD-1	AK105+Carboplatin + Pemetrexed	Phase 3	PFS
NCT05635708	PD-1	Tislelizumab + BGB-A445 or LBL-007	Phase 2	ORR
	LAG-3			
	OX-40			
NCT04547504	PD-1	Pembrolizumab or Pembrolizumab + Chemotherapy drugs	Phase 3	PFS
NCT05557591	PD-1	BNT116+Cemiplimab	Phase 1	TEAEs、SAE、ORR
	Oncology vaccines		Phase 2	
NCT04614103	TIL	LN-145	Phase 2	ORR
Clinical trials of drug resistance in immunotherapy for NSCLC.				
NCT04691817	PD-L1	Atezolizumab and Tocilizumab	Phase 1	ORR、OS
	IL-6R		Phase 2	
NCT04655976	PD-1	Docetaxel + cobolimab dostarlimab	Phase 2	OS
	TIM-3		Phase 3	
NCT03377023	PD-1	Nivolumab + Ipilimumab + Nintedanib	Phase 1	MTD、ORR
	VEGF		Phase 2	
NCT05142189	PD-1	BNT116+Cemiplimab/Docetaxel	Phase 1	DLT、TEAE、ORR
	Cancer vaccines			
NCT05195619	PEP-DC vaccine	PEP-DC vaccine + cyclophosphamide	Phase 1	Number of patients、AE、ORR
NCT03847519	PD-1	ADXS-503+pembrolizumab	Phase 1	AEs、DLTs、PFS
	Tumor neoantigens		Phase 2	
NCT04656652	TPRO-2	DS-1062a and Docetaxel	Phase 3	PFS、OS
NCT05555732	PD-1	Dato-DXd + Pembrolizumab (+ Platinum Chemotherapy)	Phase 3	PFS、OS
	TROP2			
NCT05467748	PD-1	combination of tazemetostat and pembrolizumab	Phase 1	ORR、Safety and Tolerability
	EZH2		Phase 2	
NCT05671510	CTLA-4	ONC-392	Phase 3	OS
NCT05788926	IL-12	TG6050	Phase 1	Safety and tolerability
	VEGF			

### 2.2 Immunotherapy advancements in SCLC

Based on the biological characteristics of SCLC (Hamilton and Rath, 2019; Raso et al., 2021), previous single-agent immunotherapy has been less effective when compared to NSCLC (Reck et al., 2016; Pavan et al., 2019), but in the past few years, IMpower133, CASPAIN, and ASTRUM005 have made significant breakthroughs in clinical studies (Paz-Ares et al., 2019; Mansfield et al., 2020; Cheng et al., 2022), making immunotherapy combined with chemotherapy the new standard first-line treatment for advanced SCLC. The development and utilization of immunosuppressants in advanced SCLC patients is expected to yield a substantial enhancement in their survival prognosis (Goldman et al., 2021). The CASPAIN study’s long-term follow-up findings revealed a significantly reduced occurrence of pneumonia of any grade in the group treated with Durvalumab compared to the chemotherapy group (4% vs. 7%). However, it is necessary to enhance and thoroughly assess the safety of current treatment protocols (Paz-Ares et al., 2019). Other research has indicated that PD-1 inhibitors exhibit a greater incidence of serious adverse effects compared to control groups. Furthermore, immunotherapy for advanced SCLC patients has plenty of room for improvement (Antonia et al., 2016; Pakkala and Owonikoko, 2018). The question of whether patients can continue to use immunotherapy after first-line immunotherapy combined with chemotherapy is a current research hotspot, and OS data from several clinical studies suggest that continued immunotherapy improves survival, but a large amount of evidence-based medical evidence is required (Table 2).

**TABLE 2:** Trials using combination immunotherapies for SCLC.

Trial identifier	Target	Treatment strategy	Phase	End point
NCT05228496	PD-1	Tislelizumab + Sitravatinib	Phase 2	PFS
	RTK			
NCT04501029	Topoisimerase Ⅰ	Gimatecan	Phase 2	DLT、RP2D、ORR
NCT05353257	PD-1	HLX10+carboplatin	Phase 3	OS
		Or cisplatin-etoposide + radiotherapy		
NCT04996771	PD-1	Surufatinib + Chemotherapy	Phase 1	PFS
	VEGF		Phase 2	
NCT05224141	PD-1	MK-7684A	Phase 3	OS
	TIGIT			
NCT03319940	PD-1	AMG757+Pembrolizumab	Phase 1	DLT、AEs
	DLL3			
	CD3			
NCT04453930	PD-1	Camrelizumab + Irinotecan + Platinum	Phase 2	PFS、OS
	VEGF	Camrelizumab + apatinib		
NCT05001971	PD-1	Anlotinib + Penpulimab	Phase 2	ORR
	VEGF			
NCT05815160	WEE1	Debio 0123+Etoposide + Carboplatin	Phase 1	DLTs、TEAE
NCT04539977	PD-L1	TQB2450	Phase 2	ORR
NCT04542369	PD-1	BGB-A317+Chemotherapy	Phase 2	Safety
NCT05091567	PD-L1	Atezolizumab + Lurbinectedin	Phase 3	PFS、OS
	RNA polymerase II			
	DNA synthesis			
NCT05384015	PD-1	Lenvatinib + Pembrolizumab + Chemotherapy	Phase 2	Safety and Tolerability、PFS
	VEGF			
NCT04397003	PD-L1	Neoantigen DNA vaccine + durvalumab	Phase 2	Safety and Tolerability
	Neoantigen DNA vaccine			
Clinical trials of drug resistance in immunotherapy for SCLC.				
NCT05450965	PLK1	Onvansertib	Phase 2	ORR
NCT05153239	DNA	Lurbinectedin or Lurbinectedin	Phase 3	OS
		+ Irinotecan		
NCT05296603	PD-L1	IBI-322+Lenvatinib	Phase 2	ORR
	CD47			
	VEGF			
NCT04951947	PD-1	JS201+Lenvatinib	Phase 2	ORR
	TGF-β			
	VEGF			
NCT05049863	Inosine monophosphate dehydrogenase Ⅰ/Ⅱ	MMF + Irinotecan + Allopurinol	Phase 1	AE、ORR、RP2D、DLTs
	Topoisomerase I inhibitors		Phase 2	
	ROS			
NCT05060016	DLL3	Tarlatamab	Phase 2	OR、AEs
NCT05728619	PARP1/2	HTMC0435+Temozolomide	Phase 1	DLT、AE、MTD
			Phase 2	
NCT05509699	PD-(L)1	Surufatinib + PD-(L)-1	Phase 2	PFS
	VEGF			
NCT05162196	PD-1	SBRT + Niraparib + Toripalimab	Phase 2	ORR
	PARP1/2			

## 3 Mechanisms of immunotherapy resistance

**FIGURE 2:**
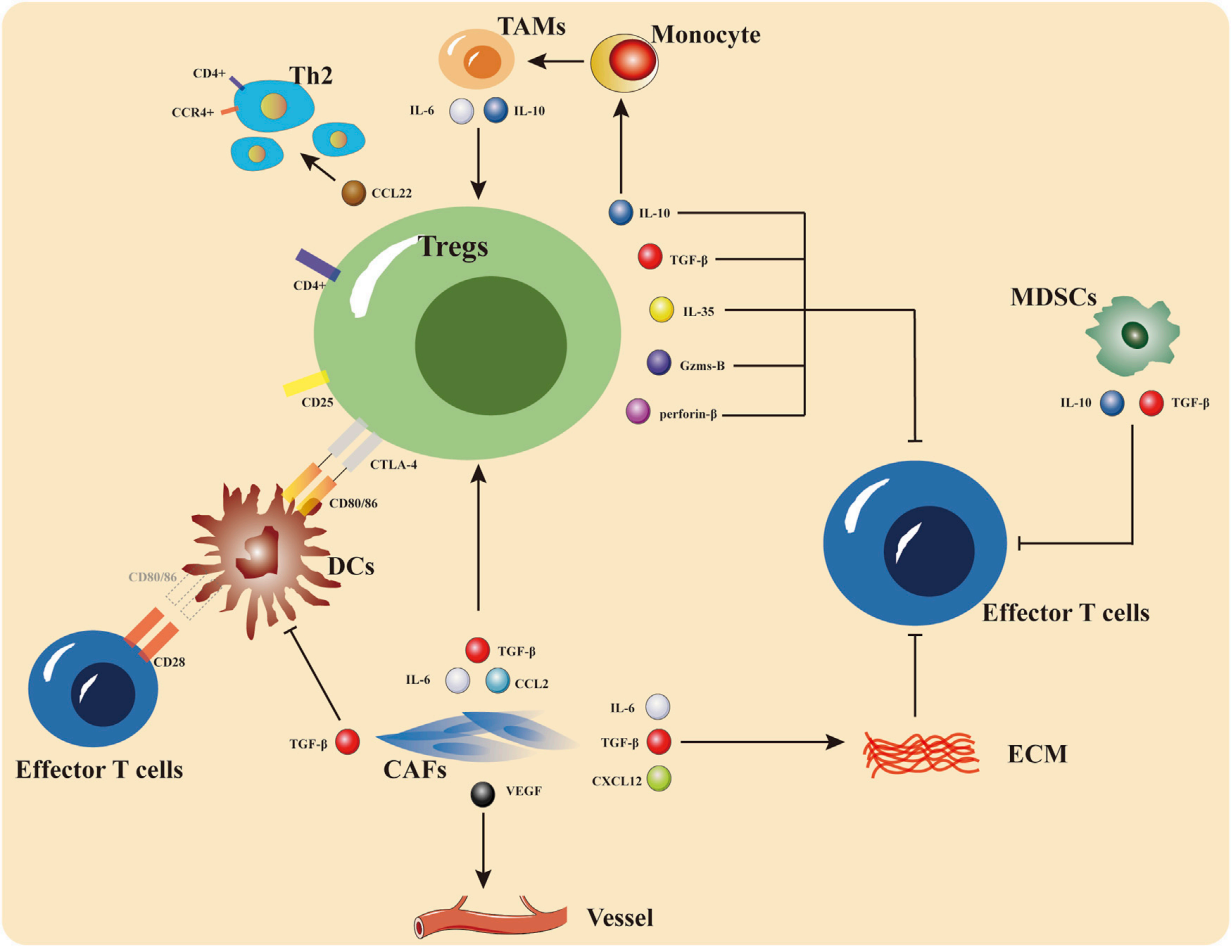
The primary site of immunotherapy resistance is the tumor microenvironment, which can be thought of as the tumor immune system’s battleground for promotion and repression. Tregs, MDSCs, and TAMs are immune-inhibitory cells that limit effector T cell function and encourage the recruitment and initiation of immune-suppressive cells by secreting cytokines including interleukin-10 (IL-10), tumor necrosis factor alpha (TNF-α), and interferon gamma (IFN-γ). ECM and CAFs are both implicated in the control of immunosuppression, according to recent studies.

Immunotherapy resistance is not yet formally defined, and the fundamental mechanisms are being intensively researched. It is possible to analyze immunotherapy resistance in terms of tumor microenvironment (TME) and internal tumor factors (Figure 2).

### 3.1 The tumor microenvironment

The TME consists of blood vessels, immune cells, fibroblasts, bone marrow-derived inflammatory cells, various signaling substances, and extracellular matrix (ECM). Previously assumed to be bystanders in carcinogenesis, these host cells and extracellular matrix components are now understood to play an important part in tumorigenesis, treatment resistance, and other processes (Belli et al., 2018; Petitprez et al., 2020; Li and Qiao, 2022). Current research indicates that the microenvironment of the tumor is the primary site of immunotherapy drug resistance and that the mechanism of drug resistance is highly heterogeneous (Passaro et al., 2020; Tang et al., 2021). A tumor’s microenvironment can influence the tumor’s metabolic and physiological functions in both positive and negative ways. Positive tumor microenvironment regulation: immune effector cells and effector cytokines.

#### 3.1.1 Positive tumor microenvironment regulation: immune effector cells and effector cytokines

The immune cells and associated matrix components recruited and/or activated by tumor cells in the TME will create a special anti-tumor inflammatory microenvironment in the early stages of tumor cell colonization or growth, thus slowing down tumor development (Belli et al., 2018; De Visser and Joyce, 2023). Immune effector cells, like CD4^+^ and CD8^+^ T lymphocytes, play a direct or indirect role in the elimination of tumor cells through the induction of channel apoptosis and/or the production of cytokines in both the innate and adaptive immune responses. B cells have received less attention in this regard, yet, there is evidence suggesting that B cells may also assume an effector function inside TME (Liu et al., 2021). Furthermore, both T cells and B cells are capable of generating memory cells that possess anti-cancer capabilities (Xiang et al., 2021; Tong et al., 2022). Increasingly, the identification of the role of DC cells in maintaining adaptive immune responses is being identified (Marciscano and Anandasabapathy, 2021; Huang et al., 2022). In addition, the TME is functionally depleted after continuous stimulation of tumor antigen and immune activation. Insufficient infiltration of effector cells, dysfunction, depletion, and impaired memory cell formation may lead to their inability to perform normal functions or even transform into a pro-cancer phenotype, thus forming an immunosuppressive microenvironment (Belli et al., 2018; Shen and Kang, 2018; De Visser and Joyce, 2023) and leading to the development of drug resistance.

#### 3.1.2 Negative tumor microenvironment regulation: immunosuppressive cells and immunosuppressive cytokines

Negative regulatory cells and cytokines make up a large portion of the immunosuppressive microenvironment surrounding solid tumor tissues. Tumor-infiltrating immune cells like Tregs, TAMs, and MDSCs also produce a significant amount of chemokines, cytokines, and proteases that block antitumor immunity and help tumors grow and spread (Tong et al., 2022; Wu et al., 2022; Yan et al., 2022). Enhancing the recruitment and penetration of TAMs, MDSCs, Tregs, and other immunosuppressive cells at tumor sites might not only improve tumor-mediated immunosuppression but also diminish tumor sensitivity to immunotherapy, which can lead to primary or acquired drug resistance (Meyer et al., 2014; Sharma et al., 2017). Tregs consist mostly of immunological subsets of CD4^+^ T cells (Paluskievicz et al., 2019). These subsets play a crucial role in suppressing the activation and proliferation of cytotoxic CD8^+^ T cells and effector CD4^+^ T cells. This suppression is achieved by reducing the expression of CD80 and CD86 on antigen-presenting cells (APCs), as well as impairing the function of both naive T cells and memory T cells. Tregs may discharge perforin and granzyme to promote the lysis of effector cells, as well as TGF-β, IL-10, and IL-35, which suppress IL-2R expression in target cells and reduce their proliferation; they can also release IL-10 to induce monocytes to differentiate into TAMs, which release IL-6 and IL-10 to activate Tregs, and they can release CCL22 to stimulate CCR4+Tregs (Paluskievicz et al., 2019; Koyama and Nishikawa, 2021).

MDSCs are bone marrow-derived cells that serve as precursors for DC cells, macrophages, or granulocytes. They are capable of inhibiting immune responses, stopping T cell and NK cell activity, which promotes tumor growth, and making tumor cells resistant to the body’s immune surveillance, which results in immune drug resistance (Birbrair, 2020; Law et al., 2020). MDSCs secrete TGF-β and IL-10, which inhibit effector T cell function, and they may enhance FOXP3+ Treg formation in malignancies like melanoma by releasing IL-10 and IFN-γ, according to multiple studies (Huang et al., 2006; Li et al., 2020). Also, CXCR2+ MDSCs are the primary subpopulation mediating immune escape in the TME of pancreatic and hepatocellular carcinoma, which can be reversed by CXCR2 antagonists (Steele et al., 2016; Wang et al., 2022).

Monocytes and macrophages in the peripheral circulation can be recruited into the tumor microenvironment, where macrophages respond to signals from tumor cells and stromal cells by changing their functional phenotype. M1-type macrophages are associated with inflammatory responses and anti-tumor immunity, whereas M2-type macrophages, which resemble TAMs, have pro-tumor properties (Gambardella et al., 2020; Boutilier and Elsawa, 2021). In a variety of cancer types, TAM infiltration is associated with poor patient outcomes. TAMs inhibit T-cell function by reducing their antigen-presenting ability and unleashing immunosuppressive factors, including IL-10 and TGF-β (Chen et al., 2019; Wang et al., 2022).

The TME contains non-immune cells that are implicated in carcinogenesis, tumor recurrences, and metastases, which are associated with resistance to treatment. ECM deposition and remodeling inside the tumor microenvironment are primarily controlled by cancer-associated fibroblasts (CAFs). Prior laboratory examinations have showcased that CAFs impede the attraction and stimulation of T cells through the secretion of CXCL12 and TGF-β. This action contributes to the deposition of ECM (Mao et al., 2021; Li et al., 2022). CAFs also release IL-6, IL-1, VEGF, and CCL2, which suppress anti-tumor immunity and promote the formation of Tregs, which are immunosuppressive. Furthermore, the CD10+GPR77+CAF subset has been found in human cancer tissue samples to contribute to tumor stem cell proliferation by secreting IL-6 and IL-8, hence contributing to tumor growth and resistance to chemotherapy (Freeman and Mielgo, 2020; Dhandapani et al., 2023). VEGF and its downstream signaling pathways can promote angiogenic conversion (Fukumura et al., 2018), and may be a significant contributor to immune resistance (Shojaei et al., 2007; Kandalaft et al., 2010; Hack et al., 2020).

#### 3.1.3 The metabolism of nutrients in the TME

To maintain the tumor’s anabolic requirements, an acidic, hypoxic tumor microenvironment is created in the TME. Metabolic changes in the TME, in turn, can impede immune cell infiltration by creating immunosuppressive metabolites, lowering the response to immunosuppression (Li et al., 2019). Glutamine, a functional substance in metabolites, produces ammonia during catabolism, which activates autophagy in immune cells (Keulers et al., 2022; Ma et al., 2022); arginine was discovered to be degraded in the tumor microenvironment by arginase, which is expressed by immunosuppressive cells such as epithelial M2 macrophages and Tregs, leading to T-cell function inhibition (Lemos et al., 2019; Azambuja et al., 2020). Elevated levels of extracellular adenosine, depletion of tryptophan, and dysregulated activation of the PI3K/Akt pathway may lead to the development of immune tolerance within the tumor microenvironment, hence diminishing the sensitivity and efficacy of immunotherapy interventions (Lemos et al., 2019; Giannone et al., 2020; Wang et al., 2020).

### 3.2 Tumor internal factors

#### 3.2.1 The activation of driver genes

Different metabolic pathways might be affected by the inheritance of particular genes. Lung cancer cells, particularly NSCLC, have a plethora of driver genes capable of mediating immune escape. Mutations, insertions, and amplifications in relevant signaling pathways and proteins, such as mutations in epidermal growth factor receptor (EGFR), anaplastic lymphoma kinase (ALK), Kirsten rats arcomaviral oncogene homolog (KRAS), and other molecular changes such as ROS1, RET rearrangement, and MET amplification, have all been linked to immunoresistance (Imielinski et al., 2012; Dantoing et al., 2021). EGFR mutations are prevalent in NSCLC (Dearden et al., 2013), and it has been shown in clinical trials that people with these mutations don't respond to immune monotherapy. This may be because suppressive cytokines are made when the EGFR signaling pathway is activated, which stops effector CD8^+^ T cells from working, and because the EGFR/GSK-3/FOXP3 axis makes Tregs multiply, which makes the immune system weaker (Borghaei et al., 2015; Wang et al., 2016). Patients with ALK fusion can decrease neoantigen formation via the PI3K-AKT and MEK-ERK pathways (Ducray et al., 2019; Hao et al., 2019) while increasing the number of immunosuppressive cells, leading to poor immunological monotherapy. Patients with KRAS mutations, on the other hand, have shown that KRAS mutations may cause immune escape by upregulating neoantigen expression and possibly PD-L1 expression without significantly activating immunosuppressive cells (Coelho et al., 2017). The immune milieu is influenced by a number of factors, and gene mutations may currently be one of the mechanisms of immunological resistance, but their clinical application needs to be explored and interpreted further.

#### 3.2.2 Tumor mutation burden (TMB)

TMB means the number of nonsynonymous mutations occurring in a particular genomic region in somatic cells. This number is typically stated as how many mutations per megabase (mut/Mb), however earlier studies also used the term directly to refer to the number of mutations (Chan et al., 2019). Several clinical phase III randomized controlled trials have shown TMB’s predictive role in efficacy (Hodi et al., 2021; Rozeman et al., 2021). Because of its ability to indirectly reflect the ability and degree of neoantigen creation. Several following clinical phase III randomized controlled trials have similarly shown TMB’s predictive role in efficacy (Tawbi et al., 2022). Tumor neoantigens are tumor-cell specific and are derived mostly from point mutations, gene insertion knockouts, shift mutations, and structural alterations in tumor cell genomes. Because of their greatest distinguishing trait, tumor cell-specific expression, they are also known as tumor-specific antigens (Schumacher and Schreiber, 2015). In clinical studies, it was discovered that a proportion of NSCLC patients treated with Pembrolizumab developed treatment resistance, losing 7–18 mutation-associated neoantigens that produce an effective response and generating complex *de novo* mutations that result in a decreased proportion of involved coding tumor antigens and altered TCR clonality, thus causing drug resistance (Anagnostou et al., 2017).

#### 3.2.3 MHC degeneration: decreased ability to present

The major histocompatibility complex (MHC), also called the major histocompatibility complex gene, is a group of highly dynamic genes that code for antigen-presenting and T-cell-activating factors that are important in immune response and control (Kumánovics et al., 2003). In 2017, a team identified HLA alleles in NSCLC patients and discovered that HLA mutations are a prevalent immune evasion mechanism in the progression of lung cancer (McGranahan et al., 2017). In NSCLC, inactivation of the B2M gene leads to improper folding and transport of MHC class I molecules to the cell surface, which results in diminished or missing expression of MHC class I molecules, rendering them unrecognized by CD8^+^ T cells and leads to immunological treatment resistance (Wang et al., 2021). Additionally, it has been discovered that TILs where drug resistance occurs exhibit deficient expression of MHC-II class II molecules (He et al., 2017). Some lung cancer cells are less immunogenic due to the lack of MHC molecules, which helps them evade immune monitoring and contributes to immunotherapy resistance.

#### 3.2.4 Epigenetic modifications

In recent years, there has been a significant focus on investigating the epigenetics of lung cancer, which has led to the discovery of the involvement of various mechanisms such as DNA methylation, non-coding RNA expression, and post-transcriptional regulation. These mechanisms have been found to play crucial roles in the development of lung cancer. Additionally, there has been a growing interest in understanding the interplay between epigenetics and the effectiveness of immunotherapy and targeted therapy in treating lung cancer (Jen et al., 2017; Huang et al., 2022). Epigenetic mutations in lung cancer are also anticipated to function as potent markers for lung cancer diagnosis, staging, and prognosis of treatment efficacy. Lung cancer development is now believed to be a protracted process that results in the progressive accumulation of epigenetic abnormalities. In turn, epigenetic alterations can promote the invasive behavior of lung cancer cells, which can be resistant to immunotherapy (Duruisseaux and Esteller, 2018).

### 3.3 Individual heterogeneity

In addition to factors in the tumor and its microenvironment, the patient’s age, weight, smoking history, gut microbial composition, the presence of other underlying diseases such as hypertension and diabetes, and previous hormone and antibiotic use may influence the effectiveness of immunotherapy (Routy et al., 2018; Knippel et al., 2021).

## 4 Reversal strategies in lung cancer immunotherapy

**FIGURE 3:**
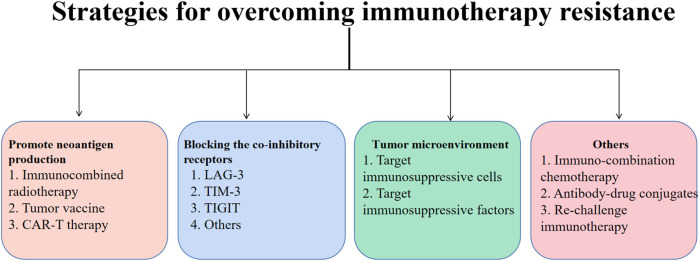
Immunotherapy resistance is common in lung cancer treatment. In this article, methods for increasing the development of tumor neoantigens, focusing on co-inhibitory receptors, and focusing on the tumor microenvironment are developed as techniques for overcoming immunotherapy resistance. Neoantigen production has been shown to be stimulated by radiotherapy, oncology vaccines, and CAR-T therapy. Inhibitors of immune checkpoints can also enhance the clinical effects of immunotherapy by targeting immune-suppressive cells and factors in the TME. Additionally, immunotherapy for lung cancer can be more sensitive when used in combination with chemotherapy other ADC medications or when given again after a pause.

Due to cancer immunotherapy resistance is the result of the collaboration of tumor microenvironment, host-related factors, and internal tumor cell factors, only a comprehensive assessment of the patient’s drug resistance status and immune status, analysis of the cause of drug resistance, and precise individualized treatment for the specific drug resistance mechanism are required. The therapy of these tumors’ internal and external factors is critical (Figure 3).

### 4.1 Immuno-combination chemotherapy

Chen Lieping’s team suggested that the lower level of immune effector cell infiltration and functional depletion led to immunosuppression and thus mediated immune escape, which led to the suggestion that based on PD-L1 expression and TIL status, TIME can be classified into four types: PD-L1-/TIL- (type I), PD-L1+/TIL+ (type II), PD-L1-/TIL+ (type III), PD-L1+/TIL- (type IV), which can provide some predictive value as well as therapeutic value for immune drug resistance. With the gradual discovery of TCR’s capacity to recognize neoantigens, a combination therapy strategy based on its clonal heterogeneity and dynamic evolution pattern, combined with different typing, can be adopted (Kim et al., 2022).

Additional research, including CHECKMATE012, IMPOWER130, and IMPOWER133, demonstrated that patients receiving chemotherapy in addition to various immune checkpoint inhibitors had much greater survival times than those receiving chemotherapy alone (Negrao et al., 2019; West et al., 2019; Mansfield et al., 2020). Nevertheless, the phase II clinical trial encompassed patients with NSCLC who had undergone chemotherapy treatment and experienced tumor progression subsequent to receiving immunotherapy in the second and third lines, and continued treatment with the immune-combination chemotherapy regimen did not result in an improvement in survival compared to chemotherapy alone. It has been postulated that the initial chemotherapy causes severe myelosuppression and that the reduction of TILs is the key to immunotherapy resistance (Cheng et al., 2022). As a result, balancing the doses and sequencing of chemotherapy and immunotherapy may be a strong method for overcoming immunotherapy resistance and improving efficacy.

### 4.2 Immuno-combination radiotherapy

It has been shown that radiotherapy can help T-cells start working by increasing MHC-I expression and making it easier for antigen-presenting cells to get rid of damaged tumor cells (Theelen et al., 2019). Radiotherapy may also encourage the creation of new antigens. Furthermore, radiotherapy can alter the tumor microenvironment, notably angiogenesis, promoting lymphocyte infiltration and thereby increasing immunotherapeutic efficacy (Wilkins et al., 2019). The PEMBO-RT study showed that combining Pembrolizumab and targeted radiotherapy improved objective remission rates in NSCLC patients (Theelen et al., 2019; 2021). According to a retrospective analysis, 208 patients with NSCLC who developed immunotherapy resistance had the best survival benefit following resistance with local therapy combined with immunotherapy compared to other regimens (chemotherapy, anti-angiogenesis, etc.). Thus, radiation combined with immunotherapy restored drug resistance to some extent, providing a novel treatment concept (Xu et al., 2021).

### 4.3 Immuno-combination immunotherapy and blocking the co-inhibitory receptors

Based on immune-mediated resistance mechanisms, blocking co-inhibitory receptors, targeting immunosuppressive factor signaling, and inhibiting interferon sustained activation signaling can increase immune efficacy. PD-1 inhibitors combined with CTLA-4 inhibitors, which have distinct mechanisms of action and can act synergistically, are the most common dual immune combination regimens (Wei et al., 2017; Rotte, 2019). When used early in the immune response, CTLA-4 inhibitors increase the number of effector T cells, while PD-1 inhibitors prevent the attachment of PD-1 to its receptor and lessen T cell depletion (Duraiswamy et al., 2013). In addition, LAG-3, TIM-3, and TIGIT are three more immune checkpoints that are gaining popularity in tumor immunotherapy research (Anderson et al., 2016; Das et al., 2017; Chauvin and Zarour, 2020). Given the strong relationship between TIGIT expression and T-cell depletion, as well as the ongoing development and clinical trials of TIGIT monoclonal antibodies, the function of inhibiting TIGIT in reversing immunological resistance remains hopeful. By attaching to MHC-II-like molecules on the surface of APCs, the LAG-3 antibody activates APCs and elicits an immune response that fights tumors. The combination of LAG-3 with Pembrolizumab resulted in an objective remission rate (ORR) of up to 38.6% in advanced or metastatic NSCLC patients that were EFGR/ALK-negative in the phase II trial TACTI-002. This ORR was much higher in patients with strong PD-L1 expression (ORR = 52.6%) (Felip et al., 2022). Therefore, it stands to reason that LAG-3 paired with ICI therapy merits additional research into how it performs in the event of immunological resistance. Another IDO1 inhibitor increased IDO1 activity in tumor cells leading to increased KYN products (Triplett et al., 2018), which suppress CD4^+^ Th1 cells, Th17 cells, CTLs, and NK cells, all of which are overexpressed in malignant tissue. However one study found that IDO1 expression in the tumor microenvironment and normal tissue in NSCLC was not significantly different (Long et al., 2019), which may be one of the reasons for the lack of clinical benefit of IDO1 inhibitors. Adenosine is abundantly expressed in the tumor microenvironment of NSCLC, where it supports the growth and differentiation of Tregs and MDSCs, increasing tumor development and metastasis (Mao et al., 2022). Moreover, treatment leading to cell death, etc. is accompanied by a large quantity of ATP release, producing high concentrations of adenosine to promote the formation of immunosuppressive line TME and further suppress immune activity. Because the therapeutic efficacy of A2aR inhibitors is still being investigated, addressing the adenosine pathway may offer promise for overcoming immunological resistance (Leone and Emens, 2018; Xia et al., 2023). Anti-angiogenesis can boost T-cell infiltration in the tumor immunological milieu, and when combined with ICI, it has also shown improved efficacy in various clinical studies, which can be investigated further to confirm the benefit of clinical application after immune resistance (Rahma and Hodi, 2019). Since prolonged IFN signaling activation causes high ligand expression of the JAK/STAT1 pathway-mediated multiple co-inhibitory receptors TCIR, resulting in T-cell depletion, and may induce tumor epigenetic and transcriptomic alterations with significant immunosuppressive effects that mediate immune resistance, JAK1 inhibition may reverse immune resistance (Kurdi and Booz, 2007). In glycolytic tumors, lactate exerts an immunosuppressive effect (Li et al., 2023). As a result of tumor cells’ Warburg effect, lactate accumulates to dangerous levels in the TME. In addition to serving as a medium for tumor development and metastasis, elevated lactate levels can also facilitate tumor proliferation and evasion of the immune system through the activation and recruitment of immunosuppressive cells and molecules. By inducing the production of vascular endothelial growth factor (VEGF) and arginase-1 (Arg1) via the HIF1-signaling pathway, lactate can promote the polarization of TAMs to the M2 subtype and assist TAMs to promote tumor growth. In addition, elevated concentrations of lactic acid in the TME may cause Tregs to develop resistance to PD-1 antibody therapy (Watson et al., 2021; Wang et al., 2022).

Other combination therapeutic options, such as targeting metabolic pathways, targeting epigenetic pathways, intestinal microbiota, and others, have emerged as new therapeutic strategies for reversing immunotherapy resistance; however, plenty of research is still being conducted because the mechanism of action is uncertain. In addition to the efficacy of immune-combination strategies for reversing immune resistance, the monitoring of side effects and safety should not be overlooked.

### 4.4 Lysovirus and CAR-T therapy

Oncolytic virus therapy involves infecting tumor cells selectively with natural or genetically modified viruses, killing and cleaving cells by virus replication, and releasing viruses to infect other tumor cells. Moreover, lytic tumor cells will produce tumor antigens, eliciting an immune response (Raja et al., 2018). CAR-T therapy, a breakthrough immunotherapy treatment, uses chimeric antigen receptors to target cancer cells. These modified T-cells recognize and attack different tumor cells when reintroduced into the patient. While, because there are currently few CAR-T cell epitopes and the recruitment rate in solid tumors is limited, current clinical studies only show significant advantages in hematological cancers (Ma et al., 2019; Sterner and Sterner, 2021). Oncolytic viruses have the potential to alleviate the problem of CAR-T not being able to infiltrate the tumor microenvironment (Evgin et al., 2022). It has been discovered that oncolytic viruses can boost the expression of CD19 on the cell surface before killing it, enhancing the cytotoxicity targeting CD19+CAR-T and achieving a higher curative impact (Park et al., 2020).

### 4.5 Tumor vaccines

Tumor vaccination stands out as a recent focal point in research. Various formats are utilized to introduce tumor antigens to patients, including tumor cells, proteins or peptides associated with tumors, genes that express tumor antigens, exosomes, and other such forms (Xu et al., 2020). With this approach, tumor immunosuppression will be overcome, immunogenicity will be boosted, the innate immune system will be activated, and both cellular and humoral responses will be triggered. The objective is to attain the aspiration of anti-tumor immunity.

### 4.6 Antibody-drug conjugates

Antibody-drug conjugates (ADCs) are a new type of medication that combines antibodies and tiny molecular cytotoxins. ADCs are composed of humanized or human monoclonal antibodies (mAbs), cytotoxic payload, and connections (Tsuchikama and An, 2018), allowing them to target and destroy cancer cells. The development of more accurate targets, the enhancement of targeted antigen-antibody binding, and the stabilization of connector coupling mode in accordance with the action characteristics of ADC drugs are the main areas of the current study. As of now, 14 ADCs are approved for use in treating advanced, recurring/refractory, and metastatic malignant cancers. More than 100 different types of ADCs are currently undergoing clinical trials (Fu et al., 2022), indicating that significant advancements have been made in the study and development of precision therapy as represented by ADC medications. This provides patients in the last line of defense against tumor survival with more treatment options and hope. The benefits of ADC alone are limited due to the evolution of drug resistance as well, so combining ADC with other anticancer medications has emerged as a key area for its drug development (Fuentes-Antrás et al., 2023). Right now, ADC-combined immunotherapy is mostly used with drugs that block PD-(L)1 and CTLA-4. Studies have shown that ADC increases antigen presentation and stimulates T-cell infiltration. It increased the immunosuppressive action of TME while promoting positive immunological modulation, achieving the impact of one plus one greater than two (Boshuizen et al., 2021). Simultaneously, researchers are looking into the potential benefits of combining ADC with other treatments, like as chemotherapy, targeted therapy, and anti-angiogenesis drugs, to provide cancer patients with even more treatment alternatives.

### 4.7 Re-challenge immunotherapy

A retrospective analysis found that the degree to which a patient was resistant to Nivolumab treatment was proportional to how well the drug worked the first time it was used (Giaj Levra et al., 2020). During subsequent administration, the utilization of an alternative PD-1/PD-L1 inhibitor has demonstrated certain therapeutic effectiveness; but the underlying rationale behind this occurrence remains unexplained. This phenomenon requires further investigation and extensive clinical evidence to understand both its cause and effectiveness.

## 5 Conclusion

Immunotherapy has given hope to lung cancer patients and resulted in long-term survival for some, however, the lack of an effective prediction method, as well as the relatively solitary and fragmented indicators, result in low predictive ability (Brueckl et al., 2020). Currently, there is no clear understanding of how the tumor microenvironment interacts with tumor cells, and clinical research data are also in the process of improvement. Clinically, there is no agreement on the definition of immunological resistance, and pseudoprogression complicates the evaluation of immunotherapy outcomes (Cheson et al., 2016; Danylesko et al., 2021). As immunotherapy gains popularity, however, medication resistance will become an increasingly pressing issue. Recently, some researchers have revealed tumor immune characteristic maps of 12 distinct types of cancers (Combes et al., 2022), providing an important notion for enhancing the relationship between tumors and immunity. However, the mechanism of immune drug resistance is diverse, and reversal strategies for various drug resistance mechanisms can improve the accuracy of customized immunotherapy targets. As a result, the treatment scheme must be chosen in conjunction with the individual tumor immunological features. It is envisaged that in the future, we will be able to create precise and tailored combined treatment plans by using programmed algorithms to assess the tumor immune microenvironment and microbiology of lung cancer patients (Jia et al., 2020). A wide range of novel therapeutic approaches can also be actively investigated and tested in an effort to improve patient survival and treatment duration, which has a promising future.
